# Impact of competitive foods in public schools on child nutrition: effects on adolescent obesity in the United States an integrative systematic literature review

**DOI:** 10.1080/16549716.2018.1477492

**Published:** 2018-06-12

**Authors:** Kirsten E. Sildén

**Affiliations:** Department of Public Health, Lund University, Ängelholm, Sweden

**Keywords:** School health policy, child nutrition, Body Mass Index (BMI), junk food, foods of minimal nutritional value, (FMNV), commercial food, vending machines, sugar-sweetened beverages, al a carte

## Abstract

**Background**: The United States (US) is currently facing a public health crisis due to the percentage of obesity in adolescents. The Center for Disease Control (CDC) stated the risks for children due to obesity are many. Adolescents obtain a large portion of their daily caloric intake at school; therefore, what foods/drinks they are consuming is so serious.

**Objective**: To identify and analyze literature on the effects of competitive foods in public schools on adolescent weight, or Body Mass Index (BMI), and possible impacts they may have on adolescent obesity in the United States.

**Methods**: An integrative systematic review of literature was conducted. The literature was collected in CINAHL, MEDLINE and EMBASE databases. Refined keyword search is further detailed in the report. Year restrictions were 2006–2017 from peer-reviewed journals and published in English, including adolescents 13–18 years old in the US. Criteria for inclusion targeted at least one of (1) sugar-sweetened beverages (SSB), (2) competitive foods, (3) commercial foods, (4) vending machines, (5) al a carte venues, and (6) school stores, examining their associations with weight measurements, using either weight or BMI, or caloric intake analysis.

**Results**: A total of 164 articles were detected and assessed, for a final analysis of 34 full text articles. Twenty-six articles met the inclusion criteria. Common aspects of interest involved BMI/Obesity/Weight (73%), (58%) examined Calorie density or consumption, (77%) discussed the Availability of competitive foods in schools, (54%) included Analysis of competitive food, beverage and nutrition policies, and (69%) addressed Other effects.

**Conclusion**: This review discovered substantial evidence that competitive foods are highly available in schools, however, lacking in robust evidence proving causality in increasing BMI or weight. There is strong corroboration in the research revealing that Other effects are factors worthy of studying further. Additional longitudinal and higher-quality research needs to be performed.

## Background

Obesity is currently a public health epidemic in the US, with more than one-third of adolescents (aged 13–18) currently weighing in as overweight or even obese [1]. BMI (weight (kg)/height (m2)) is accepted as the standard index for defining overweight and obesity due to the specificity and sensitivity it delivers [2]. It is projected that as the population becomes older, both public health and economic burdens from obesity will grow along with it [3]. The CDC reported concerning adolescent obesity, the risks for children due to obesity are numerous, including the many complications which are linked to Type 2 diabetes, including stroke, diabetic neuropathy, gastroparesis, heart disease, blindness, and even depression [4]. It is because of these increasing public health concerns that we turn our sights to the youth of America to discover why obesity is growing so rapidly and what can be done to reduce it.

Adolescents receive more than 40% of their daily caloric intake while in school, therefore, the influence of what foods and beverages they are consuming is so critical [5]. However, even with initiatives such as The Healthy, Hunger-Free Kids Act of 2010, which sets standards on competitive foods that are allowed to be sold throughout the school day for students, and the National School Lunch Program (NSLP) [6], there is still an undeniable need for further research to determine why we are still seeing a concerning increase in weight and BMI in the youth across the country.

There are many contributors to the obesity problem, with a significant amount of research on the marketing of unhealthy food towards youth and children [7–12]. Other factors include increased portion sizes, and the multitudinous snack food options offered [13].Studies on commercial and competitive foods which, according to the US Department of Agriculture (USDA), are foods available to be purchased by students which are not part of the regular school lunch programs [1] have been ongoing [5,14–28], with research thus far examining the fact that youth in schools which serve a la carte meals eat fewer healthy options, and in a school setting where soft drinks and junk foods are sold, are more likely to be obese [29].

Influences in an eating environment may directly affect food intake that could lead to overeating as well as increased risks of obesity [30]. The concern of the obesity pandemic is enough to warrant significant changes in how the US addresses issues such as ‘population-wide education, training, and motivation concerning obesity’ [31, p. 620]. Further, it is worthy to examine Foods of Minimal Nutritional Value (FMNV), which are the food choices most often available in venues of competitive food sources (a la carte, vending, and school stores) [32]. Current research also proposes that some types of carbohydrates, particularly fructose, might play a significant role in increased adiposity [33].

Hennessy et al. [26] concluded that competitive food and beverage laws within schools deserve to gain more attention as we look to addressing the obesity epidemic currently facing our nation. According to a review performed by Jaime & Lock [34, p. 52], ‘there are currently few studies which have measured the impact of school food policies on BMI’. Although adolescent obesity has been researched and discussed extensively, to my knowledge, research examining the association between availability of competitive foods in school settings and adolescent weight (BMI) has not been systematically reviewed.

The purpose of this integrative systematic literature review is to make known the literature on research which has addressed commercial foods in schools and adolescent weight (BMI), and synthesize the data to explore recommendations for further research, increase the potential for stronger health policies, and hopefully provide insight into how to better protect our youth and combat the health crisis of obesity which America is currently facing. The main aim of this thesis is to summarize the research on the effects of competitive foods in public schools on adolescent weight (BMI), and possible impact it may have on adolescent obesity in the US, by asking ‘In what ways are the weight (BMI) of adolescents impacted by competitive foods in schools?’

## Methods

To accomplish this aim, this integrative systematic review will attempt to classify quantitative peer-reviewed studies on nutrition policies in public schools which specifically address (1) competitive foods in schools and (2) the impact competitive food sources may have on adolescent weight (BMI). The methodology used in this study is an integrative systematic literature review. Data were collected through a systematic literature search.

This review will attempt to identify school-based nutrition policies of school settings which allow for competitive foods [1,32,35]. The participants in the primary studies that will be included in this integrative systematic review will be schools (public and private) in the US (Middle and High Schools) whose student population is between 13 and 18 years of age. In this review, competitive foods, often FMNV, include foods which offer less than 5% of the Reference Daily Intake (RDI) for eight selected nutrients in each serving [36], and will include SSB’s, commercial foods, vending machines, al a carte venues, and school stores.

Quantitative studies that have examined school-based nutrition policies which include or allow for competitive foods in school settings and discuss weight (BMI) and/or ways in which weight (BMI) is impacted by competitive foods in schools, were considered.

### Literature search

Cumulative Index to Nursing & Allied Health Literature (CINAHL); MEDLINE; and EMBASE databases were searched. Additional sources were examined, including bibliographies of applicable articles and relevant literature reviews, per the Methodological Expectations of Cochrane Intervention Reviews (MECIR), which advises to ‘check reference lists in included studies and any relevant systematic reviews identified’ [37, p. 16].

The screening process included scanning titles and brief abstracts initially, then full abstracts for relevance per inclusion criteria. The criteria for inclusion were: (1) peer-reviewed journals; (2) published 2006–2017; (3) English; (4) 13–18 years of age in middle or high schools; and (5) in the US. Criteria for inclusion also included at least one of the following measures: SSB’s, competitive foods, commercial foods, vending machines, al a carte venues, school stores, and their associations with weight (BMI) for adolescents in school settings. This included either actual BMI calculation, and/or caloric intake analysis. Only peer-reviewed journal articles were indexed in MEDLINE, CINHAL, and EMBASE databases. Keywords used in the review are listed in Tables 1 and 2.

**Table 1.** MEDLINE and CINAHL Prescreened Combined Search.

Search Terms	Keywords	Number of articles
S1	School OR schools	3,155,522
S2	USA OR US OR US OR USA of America	1,885,884
S3	‘food’ OR ‘food preferences’ OR ‘food habits’ OR ‘health behavior’ OR ‘feeding behavior’ OR ‘child nutrition’	1,114,267
S4	‘weight gain’ OR ‘overweight’ OR ‘BMI’ OR ‘Body Mass Index’ OR ‘Body Composition’ OR ‘Obesity’ OR ‘Adolescent Obesity’ OR ‘Pediatric Obesity’ OR ‘body weight’	927,091
S5	‘nutrition policy’ OR ‘health promotion’ OR ‘health policy’ OR ‘school health policy’	442,801
S6	‘food services’ OR ‘competitive food’ OR ‘competitive foods’ OR ‘commercial food’ OR ‘beverages’ OR ‘carbonated beverages’ OR ‘sugar-sweetened beverages’ OR ‘vending machines’ OR ‘food dispensers’ OR ‘food dispensers, automatic’ OR ‘foods of minimal nutritional value’ OR ‘FMNV’	91,114
S7	S1 AND S2 AND S3 AND S4 AND S5 AND S6	366
	S1 AND S2 AND S3 AND S4 AND S5 AND S6 sorted with Limiters: (Publication date, source type of academic journal, subject age (adolescent 13–18), English language, geography (USA, USA, Texas, Baltimore, California, Illinois, Los Angeles, Midwestern USA, Alaska, Appalachian region, Colorado, Michigan, mid-Atlantic region, New York City, Washington)	125
	*Relevant number of articles after screening abstracts from the combined search of MEDLINE and CINAHL: 73

**Table 2.** EMBASE Prescreened Database Search.

Search Terms	Keywords	Results
#1EMBASE aspect #1 obesity terms 2017–03–022017–03-021193364	‘weight gain’/exp OR ‘weight gain’ OR ‘overweight’/exp OR ‘overweight’ OR ‘bmi’/exp OR ‘bmi’ OR ‘body mass index’/exp OR ‘body mass index’ OR ‘body composition’/exp OR ‘body composition’ OR ‘obesity’/exp OR ‘obesity’ OR ‘adolescent obesity’/exp OR ‘adolescent obesity’ OR ‘pediatric obesity’/exp OR ‘pediatric obesity’ OR ‘body weight’/exp OR ‘body weight’	1,193,364
#2 EMBASE aspect #1 obesity terms 2017–03–022017–03-0215	AND [adolescent]/lim AND ‘childhood obesity’/de AND ‘adolescent disease’/de AND (‘abdominal obesity’/de OR ‘adolescent obesity’/de OR ‘obesity’/de) AND (2010:py OR 2011:py OR 2012:py OR 2013:py OR 2014:py OR 2015:py OR 2016:py OR 2017:py) AND ‘Article’/it	15
#3 EMBASE aspect #2#039 food' terms 2017–03–022017–03-021748981	‘food’/exp OR ‘food’ OR ‘food preferences’/exp OR ‘food preferences’ OR ‘food habits’/exp OR ‘food habits’ OR ‘health behavior’/exp OR ‘health behavior’ OR ‘feeding behavior’/exp OR ‘feeding behavior’ OR ‘child nutrition’/exp OR ‘child nutrition’	1,748,981
#4 and #3 EMBASE aspect #2 ' food' terms2017-03–022017-03–023896	AND ‘obesity’/de AND [adolescent]/lim AND ‘Article’/it AND (2006:py OR 2007:py OR 2008:py OR 2009:py OR 2010:py OR 2011:py OR 2012:py OR 2013:py OR 2014:py OR 2015:py OR 2016:py OR 2017:py)	3,896
#5 EMBASE aspect #3 ' US&quot; terms2017-03–022017-03–021541184	united AND states OR ‘u.s.’/exp OR u.s. OR us OR united AND states AND of AND (‘America’/exp OR America)	1,541,184
# 5 and #6 EMBASE aspect #3 ' US&quot; terms2017-03–022017-03–021761	AND ‘obesity’/de AND ‘Article’/it AND [adolescent]/lim AND (2006:py OR 2007:py OR 2008:py OR 2009:py OR 2010:py OR 2011:py OR 2012:py OR 2013:py OR 2014:py OR 2015:py OR 2016:py OR 2017:py)	1,761
#7 EMBASE aspect #4 ' school' terms2017-03–022017-03–024727654	‘school’/exp OR school OR ‘schools’/exp OR schools	4,72 7,654
#8 and #7 EMBASE aspect #4 ' school' terms2017-03–022017-03–028174	AND ‘obesity’/de AND [adolescent]/lim AND ‘Article’/it AND (2006:py OR 2007:py OR 2008:py OR 2009:py OR 2010:py OR 2011:py OR 2012:py OR 2013:py OR 2014:py OR 2015:py OR 2016:py OR 2017:py)	8,174
#9 EMBASE aspect #5 ' nutrition policy' terms2017-03–022017-03–02316924	‘nutrition policy’/exp OR ‘nutrition policy’ OR ‘health promotion’/exp OR ‘health promotion’ OR ‘health policy’/exp OR ‘health policy’ OR ‘school health policy’	316,924
#10 and #9 EMBASE aspect #5 ' nutrition policy' terms2017-03-022017-03–021187	AND ‘obesity’/de AND [adolescent]/lim AND ‘Article’/it AND (2006:py OR 2007:py OR 2008:py OR 2009:py OR 2010:py OR 2011:py OR 2012:py OR 2013:py OR 2014:py OR 2015:py OR 2016:py OR 2017:py)	1,187
#11 EMBASE aspect #6 ' competitive food' terms2017-03-022017-03–02279682	‘food services’/exp OR ‘food services’ OR ‘competitive food’ OR ‘competitive foods’ OR ‘commercial food’ OR ‘beverages’/exp OR ‘beverages’ OR ‘carbonated beverages’/exp OR ‘carbonated beverages’ OR ‘sugar-sweetened beverages’ OR ‘vending machines’ OR ‘food dispensers’ OR ‘food dispensers, automatic’/exp OR ‘food dispensers, automatic’ OR ‘foods of minimal nutritional value’ OR ‘fmnv’	279,682
#12 and #11 EMBASE aspect #6 ' competitive food' terms2017-03-022017-03–02652	AND ‘obesity’/de AND [adolescent]/lim AND ‘Article’/it AND (2006:py OR 2007:py OR 2008:py OR 2009:py OR 2010:py OR 2011:py OR 2012:py OR 2013:py OR 2014:py OR 2015:py OR 2016:py OR 2017:py)	652
Aspects #1-#6 combined with limitations	Search with limitations: Disease (obesity), Age (adolescent), Publication types (article), Publication years (2006–2017)	39
	*Results After Screening for Duplicates with Other Databases that are Relevant to Aim:17	

A search was performed in EMBASE of six aspects, resulting in 66 articles. A quick-screening through the brief abstract of articles was completed, which resulted in 39 possible articles (66 – 27 = 39). Similarly, a search was performed in EBSCO which allowed the combination of both MEDLINE and CINAHL databases to be searched simultaneously, resulting in 125 articles. Combining EMBASE results (39) with those from MEDLINE and CINAHL (39 + 125 = 164), gave a total of 164, prior to checking for duplicates (164–75 duplicates = 89).

### Screening of abstracts

**Figure 1.**
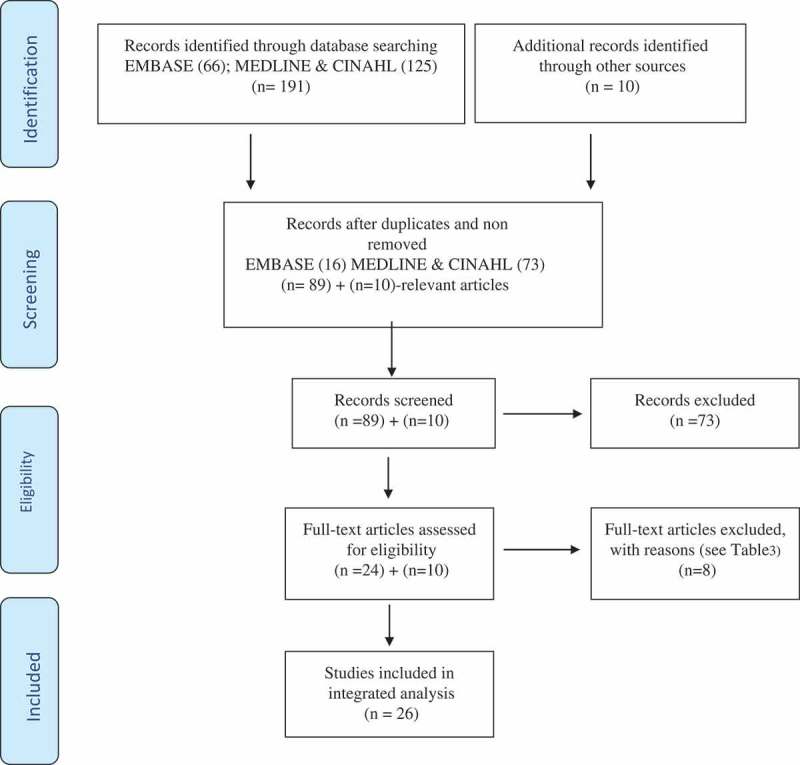
PRISMA Diagram. Moher et al. [72].

After the initial abstract screening of the 89 potentially qualified articles from all three databases (EMBASE, MEDLINE, and CINAHL) was performed, 24 articles were accepted, prior to quality check. Bibliographies were scrutinized to determine if anything more needed to be included, based on MECIR, and (10) additional articles were included (24 + 10 = 34).Additionally, according to the MECIR, ‘a PRISMA flow chart and a table of “Characteristics of excluded studies” will need to be completed in the final review.’ [37, p. 13] (see Figure 1).

### Data extraction

Further in-depth examination using a data extraction method, inspired by Urzi [38] (see Appendix 1), was utilized in narrowing down the search further, probing if the participants, settings, and interventions, are pertinent to the Key Question [39].

A pilot test of the data-extraction instrument was conducted by two reviewers, and deemed appropriate. Due to conflict in time and feasibility, the remaining 33 articles were tested by one reviewer. After utilizing the data-extraction system, and cross checking that probative information was addressed, (8) more articles were excluded with reasons (see Table 3). This left 26 articles which met inclusion criteria. Intra-rater reliability was insured through re-analysis of all data.

**Table 3.** Excluded Articles with Reasons.

References:	Reasons for Exclusion from Review:
Cisse-Egbuonye et al. [32]	Study identified the types of foods sold in vending machines and school stores in schools, discussed the availability and consumption associated with student purchase, but does not address weight/BMI/or caloric intake.
Gordon et al. [66]	Discusses availability of competitive foods in schools, school meal programs, in the prevalence of overweight and obesity. Does not address calories, BMI, or overall weight differences due to competitive foods in schools directly. Consumption, and availability, not weight.
Hawkes et al. [67]	Evaluates district policies addressing student wellness, developed an approach to determine best changes in policy to access to health food/drink in school cafeteria lunches. Does not address weight/BMI/caloric intake.
Kristensen et al. [68]	Discusses SSB taxes and impact on BMI in adolescents, but does not directly address this in school settings specifically.
Kubik et al. [20]	Discusses junk food in school vending machines and school stores and school nutrition policies, however does not address weight/BMI/caloric intake specifically. Consumption, not weight.
Schwartz et al. [69]	Discusses perceptions of weight and diet with regards to consumption of snack foods sold in schools, but actual weight, BMI, or calories were not measured or evaluated. Consumption, not weight.
Thompson et al. [70]	Primary purpose was to examine purchasing behaviors with school policy. Calculated purchasing behavior and dietary consumption, but did not address the research question on weight/BMI/or caloric intake.
Wordell et al. [71]	Study discusses school food environments, healthier school policies, and consumption and choice making of competitive foods. Does not address weight/BMI/caloric intake specifically. Consumption, not weight.

Once the screening was completed using the data analysis extraction form (see Appendix 1), the remaining 26 articles were examined more thoroughly via a more comprehensive reading of the 26 articles searching for pertinent aspects within each article which addressed the key question and criteria. The information discovery resulted in varying outcomes based on the criteria.

### Analysis

This paper confines itself to summarizing the results in an integrative analysis as the heterogeneity of the studies investigated in this review are not suitable for a meta-analysis. Haidich [40] states that systematic reviews may not need to contain a meta-analysis as there may be occasions where it is not possible or even appropriate.

### Results

The following summary of data aims to answer the objective of what impacts there are upon child weight (BMI), and/or caloric intake related to obesity when competitive foods are sold in schools, and it is the purpose of this thesis to investigate and identify any recommendations for further research as well as promote the strengthening of public health policy.

### Assessment of study quality

Based on the model presented by Ackley et al. [41], seven levels of evidence were described. Each study was evaluated based on this model, and breaking down the levels of evidence reveals that seven studies (27%) were of Evidence Level III, which include non-randomized, well-designed controlled trials; and 19 studies (73%) were Level IV, well-designed case-control or cohort studies, including cross-sectional designs. According to Detsky et al. [42], who evaluated quality strength of criteria for assigning grades of evidence, seven studies (27%) were Moderate quality, with 19 studies (73%) being of Low Quality (see Appendix 2).

### Eligible articles

The final analysis included 26 articles. The most common study designs, cross-sectional designs, were composed of 19 (73%) studies, followed by six (23%) quasi-experimental designs, and finally, one (4%) experimental study. Four interventions were accepted into the review [43–46]. The range of dates included were 2006–2017, to try to capture the most recent literature available at the time this review was completed.

All studies included adolescent age group 13–18, including Middle and High School students. Some studies included ages 6–12, and data were then extrapolated which focused on the age of inclusion for this review. The size of sample varied anywhere between 186 students involved in a high school intervention [45], to US student population age 10–17 across 50 states in the US [47].

### Synthesized presentation of research

The research synthesized is presented in Table 4, which summarizes the aspects examined most frequently within the studies. Based on Whittemore and Knafl [48], regarding methodology within integrative literature reviews, the first step in data analysis should be data reduction.

**Table 4.** Summary of Aspects Examined in Literature for Review.

Articles	Study Design	BMI/Obesity/Weight	Kcalories Consumed or measured	Availability of competitive foods in schools	Analysis of Policy	Other effects: SES/Race/Ethnicity/Sex
Anderson & Butcher [14]	Non-experimental design:Observational; cross sectional	Yes	No	Yes	Yes	No
Bauhoff, S. [51]	Non-experimental design: Observational: cohort and cross-section	Yes	Yes	No	Yes	Yes
Briefel et al. [63]	Non-experimental design: Observational; Cross-sectional	No	Yes	Yes	No	Yes
Briefel et al. [60]	Non-experimental design: Cross-sectional	No	Yes	Yes	No	Yes
Briefel et al. [61]	Non-experimental design: cross-sectional	No	Yes	Yes	Yes	Yes
Cradock et al. [43]	Quasi-experi-mental design:	Yes	Yes	Yes	Yes	Yes
Fletcher et al. [59]	Non-experimental design: Observational; Cross Sectional	Yes	Yes	Yes	Yes	No
Fox et al. [49]	Non-experimental design: Observational: cross sectional	Yes	Yes	Yes	No	No
Fox et al. [58]	Non-experimental design: Observational: cross-sectional	Yes	Yes	Yes	No	No
Hartstein et al. [44]	Non-Experimental Design: Observational; Cross-sectional	No	Yes	No	No	Yes
Hennessey et al. [26]	Non-experimental design: Observational; cross-sectional	Yes	No	Yes	Yes	Yes
Kakarala et al. [56]	Non-experimental design: Observational; Cross-sectional	Yes	Yes	Yes	No	Yes
Levy & Friend [62]	Experimental design: virtual experiment	No	Yes	Yes	Yes	No
Mendoza et al. [57]	Non-experimental design: Observational; Cross Sectional	Yes	Yes	No	Yes	Yes
Nanney et al. [47]	Non-experimental design: cross sectional; This study used quali-tative and quanti- tative methods	Yes	No	Yes	Yes	No
Park et al. [23]	Non-experimental design: Observational, cross sectional	Yes	No	Yes	No	Yes
Riis et al. [52]	Non-experimental design: Observational; Cross Sectional	Yes	No	Yes	Yes	No
Sanchez-Vaznaugh et al. [53]	Non-experimental design: Observational; cross sectional	Yes	No	No	Yes	Yes
Smith & Holloman [45]	Quasi-Experi- mental design: piloted inter-vention	Yes	No	Yes	No	Yes
Snelling et al. [46]	Non-experimental design: Observational; quasi-experi-mental	No	Yes	Yes	No	No
Taber et al. [3]	Non-experimental design: Observational: cross sectional	Yes	No	Yes	Yes	Yes
Taber et al. [54]	Quasi-experimental design: longi-tudinal study	Yes	No	Yes	Yes	Yes
Taber et al. [64]	Quasi-experimental design: observational study	No	Yes	No	Yes	Yes
Terry-McElrath et al. [50]	Non-experimental design;Observational; cross-sectional	Yes	No	Yes	No	Yes
Van Hook & Altman [16]	Quasi-experimental design: a longi-tudinal research design	Yes	No	No	No	Yes
Wiecha et al. [55]	Non-experimental design: Observational; cross-sectional analysis	Yes	Yes	Yes	No	Yes
Total		19	15	20	14	18

The themes most frequently appearing in the studies included were those examining elements of BMI/Weight/Obesity, Calorie (Kcal) Consumption, Availability of Competitive Foods in Schools, Strength of Policy, and Other Effects: SES/Race/Ethnicity/Sex.

Appendix 2 breaks down the literature components which were significant in the summary of each article accepted for this review. The breakdown consists of aim and objective, design of study, methods and data used in each study, evaluation measures, and level of evidence.

### Summary of aspects

The following is the summary of the five aspects most frequently used in the 26 articles for this review.

#### BMI/obesity/weight

Nineteen out of the 26 articles included in the review addressed competitive foods in schools based on BMI/Obesity/Weight as indicators. While the studies were diverse, several examined the associations between competitive food sources in the schools and adolescents weight [16,49,50]. Some studies explored the prevalence of childhood overweight trends before and after policies were implemented, as well as implementation and adherence to nutrition laws in schools [3,26,51–54], while others examined the interaction on junk food sold in schools along with genetic components to weight [14]. Wiecha et al. [55], debated whether there was an association between BMI and grade level with intake of SSB’s.

Several studies discussed the prevalence of competitive food and beverages in schools and causality towards increase in BMI and weight for adolescents [23,43,56]. A study analysis by Mendoza et al. [57], and Smith & Holloman [45], explored reductions in Kcal consumption by decreasing the energy density in foods and SSB’s sold in schools and possible associations with long-term weight loss and maintenance. Fox et al. [58] discussed reductions in daily Kcalories from consumption of energy-dense foods and beverages in school with possible associations to weight of adolescents. Nanney et al. [47] details the varying levels of obesity across the country in various states and regions and any relevance it has on nutrition, physical activity, and education policies within schools. Fletcher et al. [59] examined the effects of soft drink taxes on weight among children, change in BMI, obesity prevalence, or soft drink calorie consumption.

#### Kcalories consumed or measured

Articles which discussed weight through consumption of Kilocalories or Kilocalorie density were in 58% or 15 of the articles reviewed. Interventions assessed by Hartstein et al. [44], evaluated mean daily nutrient sales per student, per item in competitive food venues such as a la carte and snack bars in schools. Cradock et al. [43], measured data on consumption of SSB’s by high school students before and after policy change implementation, noting the mean per capita daily calorie consumption for students who consume SSB’s. Snelling et al. [46], compared the NSLP and competitive food offerings and purchases in students in 3 high schools, and Smith & Holloman [45], examined the impact of SSB consumption among adolescents in high school settings and the Kcalorie implications it held.

Similarly, other studies which scrutinized adolescent Kcalorie consumption of SSB’s in school settings were also performed [55,60,61], including a pilot simulation by Levy & Friend [62], who assessed SSB Kcalories consumed inside and outside of school settings, and the possible effects on an SSB tax. Kakarala et al. [56], explored the mean energy intake from competitive food sources other than a la carte in schoolchildren. Briefel et al. [63], set out to test the hypothesis that children would compensate for eating fewer SSB’s and low-nutrient, energy dense foods at school by consuming more of these items outside of school. Other cross-sectional analyzes examined data on Kcalorie consumption of competitive foods in schools before and after policy implementation or beverage tax imposed [51,57,59]. Fox et al. [49,58], examined associations between adolescent’s weight status and school food environments, and amount of Kcalories consumed by students from low-nutrient, energy-dense foods attained in school.

#### Availability of competitive foods in schools

There were 20 articles (77%) which addressed the aspect of availability of competitive foods in schools and how that related to Kcalorie consumption or weight in adolescents. Studies which correlated associations between availability, consumption patterns, and student’s BMI-related outcomes were performed by [14,26,49,50,59,60]. Whereas other studies considered the associations between a variety of competitive food sources (vending machines, a la carte, school store, snack bars) in schools with consumption of low nutrient, energy-dense foods [23,55,56,58,63].

Briefel et al. [61], and several others [3,43,52,54,62], explored change in consumption and weight status when policy interventions restricted the sale of SSB’s and and competitive food offerings on school grounds. Snelling et al. [46], sought to compare the NSLP with competitive food offerings and the impact availability and type of competitive foods had on adolescent consumption and purchasing behaviors. Nanney et al. [47], examined cross-sectional associations to discover if youth obesity prevalence and comprehensive school-based obesity prevention policies were linked. Smith & Holloman [45], analyzed the purchasing patterns of SSB consumption when restrictions on availability were implemented in school settings.

#### Analysis of policy

Fourteen studies (54%) discussed elements involving competitive food and beverage, nutrition, and physical education policies. Addressing over-weight prevalence before and after initiation of competitive food and beverage policies was observed in a number of studies [51,53,57]. Studies examined comparisons between states with competitive food and beverage policies in place versus those states with less stringent or no policies in place [3,14,26,43,63,64].

Evaluating different competitive food and beverage policies within schools around the country and their association with adolescent obesity were also examined [47,62] and, Nanney et al. [47], and Riis et al. [52], add analysis on physical education policies in addition to school nutrition policies. Taber et al [54], analyzed state school meal laws and their associations with weight status in adolescents. Fletcher et al. [59], and Levy & Friend [62], also considered effects on adding SSB taxes to predict daily Kcalorie reductions in adolescents.

#### Other effects: SES/race/ethnicity/sex

Eighteen articles (69%) addressed the element of other effects, including Socio Economic Status (SES), Race, Ethnicity, and Sex as factors of interest to the studies. Fourteen (78%) of the 18 articles which addressed other effects examined the potential impact of being in an ethnic minority group [3,16,23,26,43,4,53–55,57,61,63].

Eleven (61%) of the 18 articles were addressing SES in some capacity as being an interesting variable in the study [3,16,26,43,50,53,54,56,57,60,64]. Association with sex/gender was examined in nine (50%) out of the 18 articles when looking at other effects [14,16,23,26,43,45,51,57,63]

## Discussion

After examining all 26 articles which met all criteria for this review, it was clear that adolescent obesity is a serious public health concern. The studies analyzed and performed within this review attempted to quantify where the problems lay, with a main assumption being that since adolescents spend an average of six or more hours per day and 180 days out of each year in school settings, the school environment might hold an inimitable influence upon the diets and eating behaviors of US schoolchildren [49].

One consistent concern was the pervasiveness of competitive foods, considered FMNV in US schools [14]. Thus far, there has been scant evidence to support the associations between school food environments with eating behaviors and weight/BMI of adolescents [49]. It is with a unified consensus therefore, that all studies in this review attempted to dissect this hypothesis, with varying results. Therefore, as ‘a final step of the data analysis in an integrative review…the synthesis of important elements or conclusions of each subgroup’ …is gathered. ‘into an integrated summation of the topic or phenomenon,’ [48, p.551], which is represented in the following text.

### Positive association between competitive food and beverage policies and BMI/weight

Cradock et al. [43], found that the decline in consumption of SSB’s after the policy change in Boston Public Schools relates to approximately 45 Kcals per day, where a 45 Kcal/day reduction could potentially cut the 25% to 40% of total excess Kcalories. According to Briefel et al. [60], among students who consumed SSB’s at school, their energy intake over their entire day was approximately 229 Kcal higher than students who did not consume SSB’s at school. According to Wiecha et al. [55], the number of items purchased at school vending machines is directly associated with SSB intake and purchase.

Fox et al. [58], discovered that in middle schools and high schools, students got 171 and 219 Kcalories from low-nutrient, energy-dense competitive foods. Fox et al. [58], discovered at the middle school level, the availability of low-nutrient, energy-dense foods in vending machines in or near the foodservice area was positively associated with a higher BMI z score. And Hartstein et al. [44] noted that two Texas schools in their study showed a reduction in Kcal density from 277 to 216, however, other reductions were modest (1 to 12 Kcal per item sold). California students exposed to more stringent school nutrition policies consumed a lower proportion of their Kcalories at school, indeed, consumed less for every measure examined, compared with students in other states [64].

Results revealed that energy density significantly declined to 2.1 + 0.78 Kcal/g in the middle school study by Mendoza et al. [57]. Nanney et al. [47] found that Food Service and Nutrition (FSN) policy groupings with the strongest associations to youth obesity are policies which pertained to competitive foods and food service standards. After adjusting for factors, nutrition policies addressing competitive foods in other venues, food service director qualifications, and BMI screening remained positively and significantly related to the odds of obesity in children, as revealed by Riis et al. [52].

Levy and Friend [62], postulated that a $0.01 tax per ounce is predicted to reduce SSB consumption by 40.3–54.2 Kcal/day. Taber et al. [54], found that the unadjusted frequency of obesity was 11% higher in students who received free/reduced price lunches at school compared with students who did not purchase lunch at school. Adolescent students gained 0.44 fewer BMI units when they were exposed to specific, consistent, required competitive food laws from 2003 to 2006 than students who were not exposed to the same requirements, according to Taber et al [3].

According to Smith & Holloman [45], students who participated in their intervention reduced both their daily consumption of SSB’s as well as the number of days per week they consumed these beverages (1 serving = 150 Kcal/day). Similarly, an intervention by Snelling et al. [46], discovered that competitive food menus tend to offer foods which lack in nutrients and have higher energy densities. Kakarala et al. [56] found that the total sugar intake was higher among students who consumed competitive food/beverages when excluding a la carte items.

#### Other effects are significant

Briefel et al. [61], discovered when accounting for other effects such as age and race/ethnicity, non-Hispanic whites would save more total Kcalories than Hispanic middle school students (234 Kcal/day vs. 184 Kcal/day). Briefel et al. [63], also found that being non-Hispanic African American was associated with greater caloric intake of sweetened beverages in high school, by 42 Kcal, and that being Hispanic or non-Hispanic African American was associated with a 47 and 70 Kcal greater intake from low-nutrient, energy-dense foods, than for non-Hispanic whites, and females consumed 46 Kcal less from low-nutrient, energy-dense foods.

Bauhoff et al. [51], also discovered that there was a consistent decrease of soda consumption for females, when SSB reductions were put into place. Wiecha et al. [55], discovered that boys drank significantly more SSB’s than girls did, and that SSB intakes were higher among Hispanics vs non-Hispanics, and African Americans vs non-African Americans. Similarly, Hennessy et al. [26] noted in their study that adolescents who were overweight/obese were more often younger, have younger parents, non-Hispanic black or Hispanic, male, less vigorously active, have a TV in their bedroom, not live in a 2-parent family, and reside in a poor household. Park et al. [23] determined that in the proportions of students buying lunch from vending machines, non-Hispanic black race/ethnicity, Hispanic ethnicity, older age, and smoking were significantly higher.

Relative to other groups, fifth-grade girls in Los Angeles experienced the largest change in overweight trends, however, in the rest of California, the lower rate of increase in overweight was significant among fifth-grade boys and seventh graders [53]. In the study analyzed by Taber et al. [64], they also found that the California sample had a greater proportion of Hispanic students (76.6%) than other states that were in the sample (14.7%). However, the results were very similar when restricting the analysis to Hispanic students on consumption of caloric content of competitive foods.

Mendoza et al. [57], determined that the reduction in energy density was significantly moderated by SES, and that low-SES schools had a higher proportion of males (66%), and Hispanic students. According to Taber et al.’s [3] breakdown, it showed how states that had no 2003 laws had a relatively low proportion of students who were non-Hispanic black (9.0%) and low proportion of students in the lowest SES quintile (15.7%). However, they found that states with weak 2003 laws had a relatively high proportion of students who were Hispanic (28.0%), and who were in the lowest SES quintile (23.8%). Yet, Kakarala et al. [56] determined that competitive food use did not differ significantly between adolescents who qualified for subsidized school meals and those who did not, signifying that the cost of vended items was most likely priced so that children of any income level could buy them.

### No strong effect between school food environments on BMI/weight found overall

Anderson and Butcher [14], found a strong literature base which revealed robust correlations regarding a genetic component to weight, suggesting in their study that while school food policies have no affect on most students’ weight, policies that increase access to junk foods and SSB’s in school could be a contributing factor for those with a genetic susceptibility to weight gain. Similarly, Bauhoff et al. [51], determined there were positive effect results for obese students considering they might be more apt to purchase and consume in-school, therefore being more influenced by any nutrition policies. However, no significant statistical results regarding most student’s weights and any competitive food policies were found.

Van Hook and Altman [16], also suggest that weight during early adolescence is strongly shaped by how heavy these adolescents were in their younger years.

Fletcher et al. [59, p. 1064], discovered that ‘neither vending machine restrictions nor soft drink taxes will lead to noticeable weight reduction in children’, noting that according to their analysis, adolescents consumed just as many soft drinks in schools with vending machine restrictions as in schools without restrictions. And Hartstein et al. [44], determined that when all schools in their intervention were included, there were no significant change in number of Kilocalories sold per student between week 1 and week 6.

Briefel et al. [61], found positive results for SSB consumption in out-of-school settings versus in-school settings, suggesting that while reducing SSB consumption in schools can be beneficial (8 Kcal/day), a greater benefit would come from reducing SSB consumption outside of school settings (145 Kcal/day). And Park et al. [23], discovered no significant association in daily mean SSB consumption from vending machine on BMI status among middle school students in the US.

Nanney et al. [47], found that there was no significant correlation between Weight Assessment (WA) policies and youth obesity prevalence. Results from the analysis by Terry-McElrath et al. [50], showed that, after inclusion of the percentage of high school students eligible for free or reduced-price lunch (FRPL), all associations between student overweight and obesity and school food environment were not significant.

Van Hook & Altman [16], similarly discovered that their results demonstrated how changes in competitive foods sales in schools were not associated with changes in children’s percentile BMI. The negative relationship between obesity prevalence and school meal environment polices in high schools was constant across cross-sectional and policy change analyzes, as was determined by Riis et al. [52]. Based on the findings from Fox et al. [49], no associations between school food environments and practices, BMI z scores, or the likelihood of obesity were significant among high-school students.

### Measuring variables

A variety of different measuring variables were examined and used in each article (see Appendix 2), with some of the more common variables including 13 articles (50%) examining BMI/weight association with exposures to competitive food and beverage policies in schools, six studies (23%) compared students in school districts to those in a control group or other district. Seventeen (65%) of the 26 studies utilized questionnaires, checklists, or menu data for comparative analysis to BMI/weight/Kcal consumption, and 11 articles (42%) looked at outcome measures of per capita total Kcal consumed per day as a measuring variable. Similarly, 11 (42%) of the 26 articles analyzed in this review used comparisons within student’s eating patterns as a particularly interesting measuring variable.

### Method of analysis

Each article included in this review used one of four categories of analysis; Factor Analysis, Associations, Comparisons, and Descriptive Analysis (see Table 5). Seven (27%) of the 26 studies used Factor Analysis, whereas 11 (42%) of the articles applied an Association Analysis. Eleven studies (42%) employed Comparative Analysis, and lastly, most (16, or 62%) studies relied on Descriptive Analysis.

**Table 5.** Summary of Method of Analysis.

Articles	Factor Analysis (Clusters)	Association Analysis (multi-variate; regression; bi-variate; odds ratio)	Comparative Analysis (t-tests; comparisons; chi-Squared; correlations; ANOVA)	Descriptive statistical Analysis
Anderson & Butcher [14]		X	X	
Bauhoff, S. [51]				X
Briefel et al. [63]		X		X
Briefel et al. [61]			X	X
Briefel et al. [60]			X	X
Cradock et al. [43]		X		X
Fletcher et al. [59]			X	X
Fox et al. [49]		X		X
Fox et al. [58]			X	X
Hartstein et al. [44]	X			
Hennessy et al. [26]		X		X
Kakarala et al. [56]			X	X
Levy & Friend [62]				X
Mendoza et al. [57]			X	
Nanney et al. [47]	X			
Park et al. [23]		X	X	X
Riis et al. [52]		X		
Sanchez-Vaznaugh et al. [53]		X		X
Smith & Holloman [45]			X	X
Snelling et al. [46]				X
Taber et al. [64]		X		
Taber et al. [3]	X	X		
Taber et al. [54]	X		X	
Terry-McElrath et al. [50]	X			
Van Hook & Altman [16]	X			X
Wiecha et al. [55]	X	X	X	
Total:	7	11	11	16

### Limitations in the literature

According to this review of literature, with 26 articles included in the final analysis, each presented their limitations with regards to robustness in research. Nineteen articles (73%) were based on cross-sectional study design, which can be important in determining correlational findings, however, are not able to imply causality. It is also important to note that fifteen of the articles (58%) were based on self-reported data, while common in these types of research studies on diet and BMI/weight, could potentially alter prevalence within the data due to under or over-reporting of food and beverage consumption and purchase recall or weight/height measurements.

Five of the articles (19%) in this review mentioned that the data they were utilizing was as current as was possible to obtain, however, was still outdated and newer more vigorous research was needed. Two articles (8%) discussed the limitations in data during the pre-intervention and post-intervention phases, which would have benefited the results. Limited data collection across a variety of elements within studies was brought up in 17 of the 26 articles (65%), with most acknowledging the lack of ability to test for every factor possible to attain the strongest most unbiased results. And finally, five of the articles (19%) stated that their results were not necessarily applicable to other children, either older or in a different setting.

### Methodological limitations

There were several limitations with this integrative systematic review, one being that most of the literature available are cross-sectional studies, which are unable to define causality. Another limitation might be that only three databases were searched, therefore data was only obtained from CINAHL, MEDLINE, and EMBASE, and other literature is potentially available which was not found in this review. Generalizability is scattered, with some studies being relevant over the entire adolescent age range, and others merely pertinent to middle school, or high school aged students only. Finally, due to feasibility and time, double measures of cross-checking the data and inclusion criteria was unavailable. A pilot was done with two reviewers, yet only one researcher could complete the rest of the data extraction and analysis for this review. However, intra-rater reliability was ensured through research re-analyzation of all selected data.

## Conclusions

The results were inconclusive, with many studies unable to definitively prove that competitive food sources in the schools are causal towards BMI/weight of adolescents, and much of the literature revealing that there was indeed, no direct association between competitive foods in schools and BMI/weight. There were studies which did discover associations between weight and competitive foods sold in schools, but they were limited and did not have robust results.

Stronger policies overseeing competitive foods in schools proved to help in some significant ways, revealing that monitoring the sales and availability of FMNV in schools can only benefit students, considering how only a few Kcalories per day can make a difference long term. The most common theme throughout the literature was how even small fluctuations can create large changes in society, with easy access and availability towards unhealthy foods in schools impacting adolescents in a profound way, therefore, there needs to be an awareness and forethought regarding the future of our youth. It is apparent from the literature that policy makers need to pay extra attention to the more vulnerable groups such as those with a genetic susceptibility, lower socio-economic status, as well as examining more closely minority groups, and gender differences.

It was the definitive goal of this study to explore the literature currently available to answer the question of what ways competitive foods in school settings impacted adolescents BMI/weight. The hope was to bring to light the importance of how school environments are a strong influence and possibly a factor in the epidemic of adolescent obesity in America today. This literature review attempted to analyze the existing data systematically, thoroughly, and without bias. It is the opinion of this review that this was achieved. The literature which was examined, as well as the results of the studies analyzed, could very well be generalized to other countries other than just the US. Replicability of this study is ensured, and detailed throughout this review. Adolescent obesity is a growing epidemic worldwide, and a call for stronger research is imperative for this global public health problem.

## Paper Context

Obesity affects more than one-third of adolescents (age 13–18) in the Unites States. The economic burden and health implications are staggering. This review compiled the most recent research and examined competitive foods sold in schools to see if they are a contributing factor on adolescent obesity. This review can inform policy makers, educators, parents, and community about the obesity crisis facing our youth and the need for more rigorous research and policies to help fight it.

## Appendices

**Appendix 1.** Sample of Variables for Data Collection Table (inspired by Ursi [65]; Souza et al [38]).

1. Identification	
Title of the Article	
Title of the Journal	
Authors	
Country	United States
Language	English
Year of publication	
2. Type of publication	
Nursing publication	
Medical publication	
Publication in another area of health? Which area?	
1. Methodological characteristics of the study	
2. Type of publication	3. Research () Quantitative approach () Experimental design () Quasi-experimental design () Non-experimental design:
1. Objective or investigation question/AIM:	
2. Sample	3. Selection 4. () Random 5. () Convenience 6. () Other: 7. Size (n) 8. () Initial: 9. () Final: 10. Characteristics 11. Age: 12. Sex: M () F () 13. Race ______________________________________________ 14. Inclusion/Exclusion criteria of subjects:
15. Data Analysis	
16. Interventions performed	17. Independent variable: 18. Dependent variable: 19. Control group: yes () no (): 20. Measurement instrument yes () no (): 21. Duration of the study: 22. Methods employed to measure the intervention:
23. Evidence level and Quality based on: Ackley et al. [41] Detsky et al. [42]	

## Appendices

**Appendix 2.** Summary of the 26 articles in this Review.

Reference	Aim/ Objective	Design	Methods/ Data1. Definition of the subjects 2. Size of the sample 3. Methodology 4. Measuring variables 5. Method of analysis	Eval. Meas. Wt. BMI Kcal	Level of Evidence Ackley, et al. [41] Quality Detsky et al. [42]
Anderson & Butcher [14]	“We examine whether schools under financial pressure tend to adopt potentially unhealthful food policies and whether students’ Body Mass Index (BMI) is higher where they are more likely to be exposed to these food policies.”	Non-experimental design: Observational; cross sectional	1. Grades 1–12. 2. 451 public middle and high schools. 3. Two-sample methodology. ‘We use school food policy information from the representative sample of public schools surveyed in the SHPPS to create our proxy variable.’ 4. Three different policies: junk food availability in schools, whether schools have “pouring rights” contracts, and whether soda and snack food advertisements are allowed at schools or school events. 5. Statistical analysis; ‘our approach uses an auxiliary regression to predict exposure to a food policy, which is unobservable in the data. Standard errors were adjusted for arbitrary correlation within the state.’	BMI	Level IV; Low Quality
Bauhoff, S. [51]	“This paper evaluates the impact of an early nutrition policy, Los Angeles Unified School District’s food-and-beverage standards of 2004, using two large datasets on food intake and physical measures.”	Non-experimental design: Observational: cohort and cross-section	1. 12–13 and 14–15. 2. Middle school (206,718), High School (198,262). 3. ‘A data-driven approach was used to construct a “synthetic”’ control group that closely resembles the treatment unit in the pre-intervention period. Then generated the synthetic control group as a preprocessing step and then estimate a two-period difference-in-difference with additional individual and school level controls. The synthetic districts are constructed in three steps that were applied separately for the two datasets because of their different geographic coverage.’ 4. ‘Estimate the treatment effect using these four cohort and cross-section synthetic control groups, one for each the PFT and CHKS data, and for the cohort and cross-section samples. Since the Los Angeles beverage policy starts in January 2004, data were compared from Spring 2003 and Spring 2005.’ 5. Descriptive Statistical Analysis used.	Kcal	Level IV: Low Quality
Briefel et al. [60]	“To describe patterns of consumption of “empty calories”—low-nutrient, energy-dense foods, including sugar-sweetened beverages—by eating location among National School Lunch Program (NSLP) participants and nonparticipants.”	Non-experimental design: Observational; Cross-sectional	1. Grades 1–12. 2. 2,314 children, including 1,386 NSLP participants. 3. Twenty-four–hour dietary recalls, child surveys, and parent surveys were collected for each selected child. 4. One 24-hour recall was collected for each child and a second day’s intake on a subsample using the USDA Automated Multiple Pass Method software. 5. Descriptive Statistical Analysis used. ‘Comparisons, using t tests, of the proportion of children consuming low-nutrient, energy dense foods and beverages, mean daily energy and energy from low-nutrient, energy-dense foods, and energy density by NSLP participation status.’	Kcal	Level IV: Low Quality
Briefel et al. [63]	“To estimate the effects of school food environments and practices, characterized by access to competitive foods and beverages, school lunches, and nutrition promotion, on children’s consumption of sugar-sweetened beverages, low-nutrient energy-dense foods, and fruits/ vegetables at school.”	Non-experimental design: Cross-sectional	1. 287 schools (in 94 districts) with child-level dietary recall data; 2,314 children, grades 1–12. 2. Children aged 6 to 18 years. 3. ‘Based on the questionnaire, checklist, and menu data, we created 20 binary variables that indicated the presence of a healthful school food policy, practice, or environmental characteristic and grouped them into three domains: wellness policies and nutrition promotion practices of the district or school, competitive foods and beverages and related school practices, and characteristics of USDA lunches offered and practices related to school meals.’ 4. Food and nutrient consumption for this analysis was based on a single 24-hour dietary recall collected using the USDA Automated Multiple Pass Method software. 5. ‘Ordinary least squares regression was used to identify the association between school food environments and practices and dietary outcomes, controlling for other school and child/ family characteristics. To examine whether school food environments and practices were associated with children’s dietary outcomes at school, binary and multivariate analyzes were conducted. Descriptive analysis was conducted to estimate the prevalence of individual school food policies and practices by school type and boys’ and girls’ consumption of beverages, low-nutrient, energy-dense foods, and fruits and vegetables. Multivariate analysis was conducted to identify the relationship between school food environments and practices and dietary outcomes.’	Kcal	Level IV: Low Quality
Briefel et al. [61]	“The objective of this study was to estimate the mean calories from added sugars potentially saved by healthier beverage selections at both home and school. The study tested the hypothesis that calories saved would be greater for SSB’s than flavored milks, and that away-from-school savings would be greater than at-school savings.”	Non-experimental design: cross-sectional	1. Grades 1–12. 2. 2,314 students in grades 1 through 12 (ages 6 to 18 years), distributed among 287 schools at the elementary (n732), middle (n787), and high school (n795). 3. 24-hour dietary recalls, child/parent surveys on demographics, school meal program participation, and family eating habits were collected for each selected child in spring of 2005. 4. Descriptive analyzes of calories and added sugars from SSB’s and flavored milks by location and replacement simulation were completed using SAS, incorporating appropriate sampling weights for schoolchildren and design effects caused by the SNDA-III complex sample design. 5. Descriptive analyzes used. ‘A t test of mean differences between population subgroups was conducted to determine which groups would benefit most from beverage changes.’	Kcal	Level IV: Low Quality
Cradock et al. [43]	“The Boston Public Schools passed a policy restricting sale of sugar-sweetened beverages in Boston schools in June 2004. The objective of this study was to determine whether high school students’ consumption of sugar-sweetened beverages declined after this new policy was implemented.”	Quasi-experi-mental design:	1. Students in grades 9 through 12 (15–19). 2. 2004, 1,079 students from 17 H.S. participated; in 2006, 1,233 students from 18 H.S.’s. 3. Used data on consumption of SSB’s by H.S. students who participated in the Boston Youth Survey during February - April 2004 and February - April 2006 (N = 2,033). ‘We compared the observed change with national trends by using data from the 2003–2004 and 2005–2006 Health and Nutrition Examination adjusted for student demographics. We obtained dietary recall data from adolescents 15–19 years surveyed during the NHANES 2003–2004 and 2005–2006 periods. For analysis of national trends in consumption of SSB’s, we used the 24-hour recall interview component of the NHANES survey that documented the type, quantity, and location of each beverage consumed, researchers coded beverage items according to the (USDA) Food and Nutrient Database.’ 4. The outcome measure of per capita total k/cal consumed per day SSB’s included consumption of soda, sport drinks, fruit drinks and punches, low-calorie drinks, sweetened tea, and other sweetened beverages. 5. Descriptive Statistical Analysis. ‘We used linear regression analysis to examine changes in mean servings per day of SSB’s between 2004 and 2006, adjusting for potential differences in student composition.’	Kcal	Level III: Mod. Quality
Fletcher et al. [59]	“In this paper, we outline important concepts related to these policies. We present new empirical evidence of the likely effectiveness of school vending machine restrictions and increasing taxes on soft drinks on reducing obesity rates in children. We conclude by discussing how the effectiveness of these policies might be increased.”	Non-experimental design: Observational; Cross Sectional	1. 5th (2004) −8th grades (2007). 2. None provided. 3. ‘Using data on soft drink consumption and vending machine restrictions from the nationally representative ECLS-K. We focused on the 5th grade (2004) and 8th grade (2007) survey waves, to measure soft drink consumption and accessing data from (NHANES) III (1988–1994) and IV (1999– 2006). Individual foods and beverages from the dietary recall were coded and classified using the USDA’s Survey Nutrient Databases. We examined the effects of soft drink taxes on soft drink consumption and weight among children and adolescents using data from (NHANES) III (1988–1994) and IV (1999– 2006).’ 4. Soft drink consumption and accessing data; Indiv. foods and beverages were coded and classified; soft drink consumption and weight were examined. 5. Descriptive Statistical Analysis used. Comparisons used for BMI levels across ages and sexes.	BMI	Level IV: Low Quality
Fox et al. [49]	“To examine the association between school food environments and practices and children’s body mass index (BMI; calculated as kg/m2). In this article, we examine associations between school food environments and practices and children’s weight status, as measured using BMI.”	Non-experimental design: Observational: cross sectional	1. Grades 1–12. 2. 287 schools and 2,228 children. 3. 287 schools (in 94 school food authorities) in which site visits were completed to inventory competitive food sources and collect child-level data, and 2,228 children who had valid height and weight data; Foodservice managers completed a menu survey that provided information about the foods offered in school meals each day during a specified “target week.” 4. Students and parents completed brief interviews that collected data on sociodemographic characteristics of the child’s household, as well as information about the child’s level of physical activity, the amount of time the child spent watching television or videos, using the computer, or playing video games, recent dieting behavior, and parental perceptions about how the child’s usual food intake and activity level compared to other children of the same age and sex. Sampled children were measured and weighed. 5. Descriptive Statistical Analysis used. ‘Ordinary least squares regression was used to estimate the associations between school food environments and practices and BMI z scores and logistic regression was used to estimate associations between school food environments and practices and the likelihood of obesity.’	BMI	Level IV: Low Quality
Fox et al. [58]	“To describe the availability of competitive foods in US public schools, consumption of competitive foods by children, and contributions of competitive foods to energy intakes.”	Non-experimental design: Observational: cross-sectional	1. Grades 1–12. 2. 287 schools and 2,314 children. 3. SNDA-III data were collected between January and June 2005. Food service managers in each sampled school completed a menu survey that provided information about the foods offered in school meals each day during a specified “target week. Trained field interviewers completed observation checklists that documented the competitive food sources available in each school and the specific types of food that were offered. Three separate checklists were completed to document competitive foods available in each school from three major sources: foods available for à la carte purchase in the cafeteria; vending machines; and other on-campus food sources. Observations were conducted on one randomly selected day during the time interviewers were at the school to collect 24-hour dietary recalls from children. Interviewers also identified all of the specific locations within the school where children could obtain foods and beverages and assigned a separate code to each location. Interviewers used information provided by the food service manager to rank each location on the relative availability of competitive foods. Rankings indicated that all, most, about half, a small amount, or none of the foods sold/served at the location were part of subsidized school meals. Sampled children were interviewed in person. 4. Foods available for à la carte purchase in the cafeteria; vending machines; and other on-campus food sources; location of food sources; 24-hour recall interviews. 5. Descriptive Statistical Analysis used. ‘Most analyzes were limited to estimation of means and proportions. Two-tailed t tests were used to test the significance of differences between children who did and did not eat a school lunch.’	Kcal	Level IV: Low Quality
Hartstein et al. [44]	“Document whether a 6-week snack bar/a la carte line intervention improved kilocalories, macronutrients, and food offerings purchased per student and per item sold (nutrient density) compared to baseline.”	Non-Experimental Design: Observational; Cross-sectional	1. Students grades 6–8. 2. 6,248 students. 3. Reducing all reg. chips serv. size bags to <1.5 oz., increase lower-fat chip offerings by 25%; offer bottled water in a 20-oz. size, and limit all sweetened beverages to < 12 oz. 4. To assess if nutrient density of items sold was improved by the intervention, kilocalories, fat, and % fat from items sold per day were divided by the total number of items sold that day. 5. Statistical analyzes used. ‘Mixed models, accounting for the clustering of observations within schools, were used to analyze changes between baseline and week 6 for two models.’	Kcal	Level IV Low Quality
Hennessy et al. [26]	“This study attempted to determine whether state laws regulating low nutrient, high energy-dense foods and beverages sold outside of the reimbursable school meals program (referred to as ‘‘competitive foods’’) are associated with children’s weight status.”	Non-experimental design: Observational; cross-sectional	1. 11–14. 2. 16,271 interviews. 3. ‘The National Cancer Institute’s Classification of Laws Associated with School Students (CLASS) database was used to evaluate the stringency of codified school nutrition laws for each of the 50 states and the District of Columbia. Competitive food laws were scored in relation to the Institute of Medicine standards.’ 4. Independent variable: a composite score of competitive food and beverage laws for middle schools. Dependent variable: overweight and obesity weight status defined as (BMI). 5. Descriptive Statistical Analysis used. Bivariate and multivariate logistic analyzes performed.	BMI	Level IV: Low Quality
Kakarala et al. [56]	“Excluding a` la carte items from competitive foods, the objectives were to: (1) assess competitive food use by gender, ethnicity, eligibility for free or reduced-price meals, and participation in school meals programs, (2) determine differences between grade levels in energy intakes obtained from food sources, (3) determine the nutrient intake derived from competitive foods for students who consumed them, and (4) determine energy-adjusted differences in 24-hour nutrient intakes of protein, calcium, iron, & other selected nutrients betwn compet. food consumer and non-consumers.”	Non-experimental design: Observational; Cross-sectional	1. Grades 1–12. 2. 2309 students. 3. ‘Competitive foods/beverages use, excluding a la carte items, was examined using the third School Nutrition Dietary Assessment Study (SNDA III), a nationally representative sample of 2309 schoolchildren in grades 1 to 12. Trained interviewers administered questionnaires to obtain 24-hour food recall data on a school day. All students of middle schools and high schools self-reported their dietary intakes.’ 4. (SNDA III). Data on food source, Competitive foods/beverages use, dietary interview data were collected, reported food items to food composition data; gender (male, female), ethnicity (non-Hispanic white, non-Hispanic black, Hispanic, other), income eligibility for NSLP/SBP (eligible for free meals, eligible for reduce-price meals, not eligible), and participation in NSLP/SBP. 5. Descriptive statistical analysis used. Analysis of variance techniques were employed to test significance of differences between groups while controlling for covariates.	Kcal	Level IV: Low Quality
Levy & Friend [62]	“In this paper, we develop a simulation model of the pathways of policy effects of school-based access to nutrition, school-based education and SSB tax policies on the consumption of SSB’s by youth, and the resulting effects on caloric intake. The model will embody a systematic approach that considers the effect of various school nutrition policies on the number of SSB calories consumed inside and outside of school, and how changes in SSB consumption affect the consumption of other foods (more specifically, their associated caloric intake).”	Experimental design: virtual experiment	1. Review of literature and policies. 2. None provided. 3. ‘We reviewed the literature on the direct effects of school nutrition policies on SSB consumption, the effect of price on SSB consumption (with a focus on youth), the effects of reduced consumption of SSB’s in school on the consumption of other foods in school (LNEDs or otherwise), the effects of SSB consumption in school on the consumption of SSB’s and other foods outside of school, and the effects of SSB consumption on caloric intake (both through direct reductions in the amount consumed and the effect on the consumption of other foods).’ 4. ‘We apply those estimates where feasible in our current modeling approach and incorporate new studies conducted since that review. We limit the analysis to the effect of SSB policies on overall caloric intake.’ 5. Statistical Descriptive Analysis used.	Kcal	Level II-III; Mod. Quality
Mendoza et al. [57]	“The study’s objective was to assess the impact of the Texas Public School Nutrition Policy on children’s energy density by using a pre- and post-policy evaluation. Specifically, the research objectives were to examine the changes in energy density after the policy, determine whether socioeconomic status moderated the impact of the policy on energy density, identify changes in the contribution of individual food groups to energy intake after the policy was implemented, and determine whether socioeconomic status mod. changes by individual food groups to energy intake.”	Non-experimental design: Observational; Cross Sectional	1. 6–8th grade students. 2. 2,616 students 2001–2002; 10,172 students 2005–2006. 3. 6–8th grade students completed anonymous lunch food records immediately after lunch in the cafeteria. ‘On each school day during the week, research assistants selected either one or two tables of students at each lunch period and asked students to complete a food record for lunch consumption only. Lunch tables were selected starting at one side of the cafeteria and then on subsequent days, tables closer to the opposite side of the cafeteria were selected until all tables had been sampled. This process was then repeated throughout the entire school year, September through May. Research staff did not collect data on refusals.’ 4. ‘Graphic data were collected in year 1. From the food records, data were entered into the Nutrition Data System to obtain average daily lunch intake of calories and the average gram weight of food at lunch.’ 5. Analysis of variance (ANOVA) was used to address the study objectives. Two-way ANOVAs, with year and (SES) as factors, were used to identify differences in energy density between academic years and school SES. To control for inflated type I errors caused by multiple testing, the Bonferroni correction was used and the significance level was set to 0.0167.	Kcal	Level IV: Low Quality
Nanney et al. [47]	“The primary aim of this paper was to develop a comprehensive evaluation approach to describe the policy environment related to school-based obesity prevention efforts in each of the 50 US states. A secondary aim was to examine the cross-sectional associations between current state policy environments and youth obesity prevalence.”	Non-experimental design: cross sectional; This study used quali-tative and quanti- tative methods	1. Ages 10 –17. 2. US student populations 10–17 across 50 states in the US 3. ‘Using 2006 (SHPPS) state data, qualitative and quantitative methods were used (2008 –2009) to construct domains of state-level school obesity prevention policies and practices, establish the validity and reliability of the domain scales, and examine their associations with state-level obesity prevalence among youth aged 10 –17 years from the 2003 National Survey of Children’s Health’ 4. Used state-level data from SHPPS 2006. scaled scores were developed for all state-level obesity-related policies and practices, for areas of FSN, PAD, and WA. 5. Statistical Analysis. To manage the extensive SHPPS policy data set, investigators used principal component analysis (PCA) to create an initial set of policy clusters.	Wt.	Level IV: Low Quality
Park et al. [23]	“This cross-sectional study examined the prevalence of students buying snacks or beverages from school vending machines instead of buying school lunch and predictors of this behavior. the purpose of this study was to examine the prevalence and behavioral predictors of students in middle school who purchase items from school vending machines instead of purchasing a traditional cafeteria lunch.”	Non-experimental design: Observational, cross sectional	1. Grades 6–8. 2. 4,322 students. 3. Students were asked, “During the previous five school days, how many days did you buy a snack or beverage from the vending machine instead of buying lunch?” 4. Analyzes were based on the 2003 YPANS using a representative sample of 4,322 students in grades 6-8 in 73 Florida public middle schools. Student and school characteristics were examined. Data were weighted by sex, grade, and region to represent all Florida public middle school students in grades 6–8. Outcome measure was buying a snack or beverage from vending machines 2 or more days during the previous 5 days instead of buying lunch. 5. Statistical Analyzes included Chi-Squared tests and logistic regression. Descriptive statistics were expressed as proportions. Chi-Squared tests were used to examine differences within categories. Odds ratios and 95% confidence intervals (CIs) for risk factors for buying lunch from vending machines were calculated using multivariable logistic regression. Interactions between exposure variables were also examined.	BMI	Level IV: Low Quality
Riis et al. [52]	“The objective of this study was to examine relations between state-level school policies and childhood obesity for youth ages 10–17 years.”	Non-experimental design: Observational; Cross Sectional	1. Students grade 5–12. 2. Children aged 10–17. 3. Wilcoxon signed rank sum tests were used to examine the extent of change in state nutrition and PE policies from 2003 to the most recent assessment (2006 for nutrition and 2007 for PE policies). Tests were also used to compare the comprehensiveness of the most recent nutrition and PE policy domain scores across school-type (ES, MS and HS). 4. 11 nutrition and 5 physical education (PE) domains were examined for elementary (ES), middle (MS), and high school (HS) children. 5. Bivariate logistic regression examined the relations of each independent variable, including individual, family and neighborhood factors, with the odds of being obese.	BMI	Level IV: Low Quality
Sanchez-Vaznaugh et al.[53]	“This is one of the first studies examining the postulated population-level influence of recently implemented policies aimed at sales of competitive foods and beverages in schools.”	Non-experimental design: Observational; cross sectional	1. Fifth and seventh graders. 2. 475,389 (2001); 602,567 (2008) 3. ‘The Fitnessgram database includes information on children’s (BMI) age, sex, race and ethnicity, grade, and physical fitness test results. To account for school- and district-level characteristics, we merged 2001–2008 Fitnessgram data with school and district data from the California Department of Education’s databases and 2000 census data.’ 4. BMI values were compared to the (CDC’s) 2000 growth charts. ‘Student-level explanatory variables included age, race and ethnicity, and physical fitness. characteristics of the students, schools, and districts, overall and by year, for the LA Unified School District and the rest of California were measured separately.’ 5. ‘We estimated the characteristics of the students, schools, and districts, overall and by year, for the LA Unified School District and the rest of California separately, using means and standard deviations or frequencies as appropriate. We estimated adjusted overweight prevalence for each study year and the rate of change, or trend, of overweight prevalence using multilevel multiple logistic regression models.’	BMI	Level IV; Low Quality
Smith & Holloman [45]	“The purpose of the project was to examine school-based purchasing patterns of SSB and explore the impact of a school-based and student-led intervention aimed at limiting short-term and longer-term SSB consumption. To understand SSB consumption patterns, both daily servings and the numbers of days per week that SSB were consumed were examined.”	Quasi-Experi- mental design: piloted inter-vention	1. Grades 9–12. 2. 186. 3. The piloted intervention consisted of the creation of a TAC at each of 2 high schools to design specific components of the intervention: a ‘‘Sodabriety’’ 30-day challenge. Each TAC consisted of 12 members overall: 2 teachers and at least 2 students from each grade in school (grades 9–12). To gather baseline data, pre-intervention an assessment of school vending machine beverage choices was completed. Data was collected at 3 time-points: pre-intervention, immediately post-intervention, and at 30 days’ post-intervention. Recruitment for the ‘‘Sodabriety’’ project occurred in 2 phases. (1) focused on recruitment of members to serve on each TAC; (2) focused on recruitment of students attending each school to participate. 4. Demographic survey. A Vending Machine Survey was designed and completed by study personnel prior to implementation of the intervention. A 10-item beverage survey was completed by each participant at 3 times: preintervention, immediately post-intervention, and 30 days’ post-intervention. Each participant was provided a beverage log to complete during the 30-day intervention. 5. Descriptive variables were collected by self-report during the pre-intervention data collection. Data were analyzed using both descriptive and inferential methods. Descriptive data were analyzed by calculating frequency, means, standard deviations, and ranges of values. Paired t tests were conducted.	Kcal	Level III: Mod. Quality
Snelling et al. [46]	“The aim of this study was to compare the NSLP and competitive food offerings with the food purchased at 3 public high schools, collectively.”	Non-experimental design: Observational; quasi-experi-mental	1. High School students (grades 9–12). 2. 5249. 3. ‘Over a 4-week cycle, daily food purchases were gathered and the proportions of green, yellow, and red foods offered and purchased was compared. Competitive food items were coded according to nutrient information obtained from the food label. Purchasing data for both competitive and NSLP food items was collected each day by the food service staff at 3 public high schools.’’ 4. The food items were coded by a system based on the Stoplight Diet for analysis, placing food in 3 categories: green, yellow, or red. 5. Statistical Descriptive Analysis.	Kcal	Level III: Mod. Quality
Taber et al. [64]	“To determine whether nutrient intake is healthier among high school students in California, which regulates the nutrition content of competitive foods sold in high schools, than among students in states with no such standards.”	Non-experimental design: Observational: cross sectional	1. Grades 9–12. 2. 680 high school students. 3. State laws governing fat, sugar, and nutritional content of competitive foods sold in vending machines, school stores, and à la carte venues were studied. Student data on nutrient intake were obtained from the (NYPANS), conducted by the (CDC). 4. Estimated differences in (1) total caloric intake, overall and stratified by location of consumption; (2) proportions of total calories consumed in the forms of protein, carbohydrates, and different types of fat, and (3) total intake of macronutrients and micronutrients. 5. General linear models were used to estimate differences in average dietary intake between students in California and states that did not have laws regarding the nutrition content of competitive foods.	Kcal	Level IV; Low Quality
Taber et al. [3]	“To determine if state laws regulating nutrition content of foods and beverages sold outside of federal school meal programs (“competitive foods”) are associated with lower adolescent weight gain.”	Quasi-experimental design: longi-tudinal study	1. Middle school’s grades 5th −8th. 2. 6300 students. 3. The Westlaw legal database identified state competitive food laws that were scored by using the (CLASS) criteria. States were classified as having strong, weak, or no competitive food laws in 2003 and 2006 based on law strength and comprehensiveness. Objective height and weight data were obtained from 6300 students in 40 states in 5 and 8th grade (2004 and 2007). 4. ECLS-K used. General linear models estimated the association between baseline state laws (2003) and within-student changes in BMI, overweight status, and obesity status. Identity measures were examined: gender, age, race/ethnicity (non-Hispanic white, non Hispanic black, Hispanic, non-Hispanic other), socioeconomic status (SES) 5. ‘Fixed-effect models estimated the association between law changes during follow-up (2003–2006) and within-student changes in BMI and weight status. A robust SE was used to account for within-state clustering.’	BMI Kcal	Level III: Mod. Quality
Taber et al. [54]	“To determine if state laws with stricter school meal nutrition standards are inversely associated with adolescent weight status, while controlling for unmeasured state-level confounders.”	Quasi-experimental design: observational study	1. 8th grade students in 40 states. 2. 4,870 students. 3. ‘Codified state statutory and administrative laws governing school meals were collected as part of the Bridging the Gap research program.’ Student data from the (ECLS-K). Other ECLS-K data in this study was gotten from interviews with parents/guardians and questionnaires done by students. 4. Independent variable: Type of School Lunch they usually obtained. Dependent variable: primary outcomes of interest were (BMI) percentile and obesity status. Control group: Students who do not obtain lunch at school 5. Statistical Analysis. ‘All analyzes used generalized estimating equations with an exchangeable correlation matrix and identity link to control for state-level clustering’.	BMI	Level III: Mod. Quality
Terry-McElrath et al. [50]	“Identify trends in the availability of various food choices in USA’ middle and high schools from 2004 to 2007, and examines the potential associations between such food availability and students’ self-reported eating habits and body mass index (BMI)–related outcomes.”	Non-experimental design Observational; cross-sectional	1. Students 8th, 10th, and 12th grades. 2. 45,000 students interviewed each year/ 2004 to 2007. 3. Report uses data from two studies: Monitoring the Future (MTF), and Youth, Education, and Society (YES). 4. Independent variables were all measured at the school level, and included: types of foods offered, locations such foods could be obtained, if the school provided breakfast, and school start time. 5. Trend analyzes were conducted at the school level using the Mantel-Haenszel c2 test for significance. Multivariate analyzes were conducted to assess the association between the school food environment and student food consumption, with correction for design effects resulting from clustered sampling using the survey logistic procedure.	BMI	Level IV Low Quality
Van Hook & Altman [16]	“We use longitudinal data to estimate the association between the introduction of competitive food sales in children’s schools and weight gain from fifth grade to eighth grade, and we exploit variation in the timing of the transition from elementary to middle school to estimate exposure to competitive foods in middle school environments. Second, we assess whether the estimated associations of competitive foods with weight gain vary significantly by gender, race/ethnicity, and family socioeconomic status (SES).”	Quasi-experimental design: a longi-tudinal research design	1. Fifth and eighth grades. 2. 19,450 children who attended school in the same county in both fifth and eighth grades. 3. ‘In the 5th and 8th-grade waves of data collection, school administrators were asked a series of questions about the availability of competitive foods for students to purchase. Children were also asked the types of food available for purchase at school. For both 5th and 8th grades, we created a dichotomous measure of competitive food sales for schools that sold food through at least one competitive food venue (i.e. vending machines, snack bars, or a` la carte). We conducted supplementary analyzes of the types of food sold, using two competitive food scales.’ 4. ‘Height and weight measures were collected from the children during the spring and fall of kindergarten and 1st grade and during the spring of 3rd, 5th, and 8th grades, which were converted to percentile BMI in accordance with CDC guidelines. We used data from the of (ECLS-K) 1998–1999.’ 5. Used estimate fixed effects models, which model changes in children’s weight as a function of changes in competitive foods. Descriptive statistical analysis used.	BMI	Level III- Level IV; Mod. Quality
Wiecha et al. [55]	“To examine associations between use of school vending machines and fast-food restaurants and youth intake of sugar-sweetened beverages.”	Non-experimental design: Observational; cross-sectional analysis	1. Middle School grade 6–7. 2. 1,474 students. 3. ‘From a group randomized obesity intervention, we analyzed baseline data from 1,474 students in 10 Massachusetts middle schools with vending machines that sold soda and/or other sweetened drinks. Students completed a self-report survey of 83 questions during a single class period in Fall 2002 administered by trained study staff. Vending machine presence, availability to students, and contents were determined in Spring 2003 by study staff using a standardized data collection instrument.’ 4. ‘Daily SSB consumption (regular soda, fruit drinks, and iced tea), purchases from school vending machines, and visits to fast-food restaurants in the preceding 7 days were estimated by self-report. Heights and weights were obtained by school nurses standardized in measurement techniques by study staff. BMI was generated using an age- and sex-specific algorithm from the CDC.’ 5. Statistical analyzes performed and nonparametric tests were performed on unadjusted data; multivariable models adjusted for sex, grade, body mass index, and race/ ethnicity, and accounted for clustering within schools.	BMI	Level IV: Low Quality
